# INHBA is a novel mediator regulating cellular senescence and immune evasion in colorectal cancer

**DOI:** 10.7150/jca.61556

**Published:** 2021-08-13

**Authors:** Shuai Chen, Yu Gong, Yu Shen, Yu Liu, Yue Fu, Yi Dai, Adeel ur Rehman, Liming Tang, Hanyang Liu

**Affiliations:** 1 Center of Gastrointestinal disease, The Affiliated Changzhou No. 2 People's Hospital of Nanjing Medical University, Changzhou, Jiangsu 213000, P.R. China.; 2 Department of Hepatology & Gastroenterology (CVK), Charité Universitätsmedizin Berlin, D-13353 Berlin, Germany.; 3 Cell Biology, Deutsches Rheuma-Forschungszentrum Berlin (DRFZ), a Leibniz Institute, Berlin, Germany.; 4 Institute of Radiology, Charité - Universitätsmedizin, D-13353 Berlin, Germany.

**Keywords:** INHBA, Cellular senescence, Immune evasion, Immune checkpoints, Colorectal cancer

## Abstract

Colorectal cancer (CRC) is one of the most mortal cancers in the world. Multiple factors and bio-processes are associated with in tumorigenesis and metastasis of CRC, including cellular senescence and immune evasion. This study aims to identify prognostic and immune-meditating effects of INHBA in CRC. Microarray datasets were downloaded from the Gene Expression Omnibus (GEO) database to screen the differentially expressed genes (DEGs) in senescent cells and CRC tissues from the Cancer Genome Atlas (TCGA). Key factor was settled from the alternative DEGs set. Enrichment analyses and functional networks prediction were determined from online databases. Correlation analyses were performed to reveal the association among key factor, immune infiltration, T cell biomarkers and immune checkpoints. Moreover, expressions of key factors and immune checkpoints of tissue and blood samples from CRC patients as well as human CRC cell lines were measured. Results showed that Inhibin beta A (INHBA) was sorted out as a senescence-related factor and a prognostic predictor in CRC. What's more, INHBA was found highly co-expressed with T-cell biomarkers and immune checkpoints. In conclusion, INHBA was considered as a senescence-related regulator and a prognostic predictor in CRC, which also mediating immune evasion.

## Introduction

Colorectal cancer (CRC) constitutes about 10% of all cancers and cancer-related death diagnosed around the world every year 1. Risk factors for colorectal cancer are relatively complex, and epidemiological studies showed that the gene mutation, unhealthy lifestyle, obesity and environmental factors are recognized as potentially carcinogenic factors 2. The combined adjuvant chemotherapy and surgery are common treatment employed to CRC, which benefits less in patients at middle-late stage. In addition, the increasing incidence of chemotherapy drug resistance dramatically influences the curative effect of traditional chemotherapy 3.

Immune evasion and immune checkpoints are associated with the tumor immunotherapy research. Theoretically, [T cell receptors (TCR)] - [major histocompatibility complex (MHC)] binding bio-process are regulated by co-stimulation or co-inhibition of signals that tumor cells use to evade immune attack 4. Currently, there are two major types of immune checkpoint inhibitors (ICIs) applied to clinical practice [programmed death protein-1/ligand-1 (PD-1/PD-L1) inhibitors and cytotoxic T lymphocyte antibody 4 (CTLA-4) inhibitors]. Although ICIs can prevent T cell dysfunction and apoptosis and enhance cytotoxic killing effect on tumor cells, long-term exposure to persistent antigens and inflammation lead to excessive infiltration of T cells in the tumor cell microenvironment and continuous stimulation. The depleted T cells gradually lose their effector function, which generates T cell exhaustion 5, 6. Both overexpression of immune check-points and T cell exhaustion can promote immune evasion, as well as reduce the efficacy of tumor ICIs therapy.

Senescence represents a series of degenerative behaviors in tumor tissues, which contain growth stagnation, manifested by phenotypic changes such as chromatin remodeling, metabolic reprogramming, and morphological changes 7, 8. Senescence associated secretion phenotype (SASP) is one of the most significant characteristics of senescent cells, which promotes immune cell infiltration and mediates inflammatory response by releasing large amounts of cytokines, chemokines, growth factors and proteases, and regulates the cellular immune microenvironment 9. In addition, SASP regulates the tumor microenvironment through a variety of signaling pathways, enabling the tumor to escape the destruction of foreign factors such as immune attacks 10, 11. Gewirtz's group reported on 'Cancer Research' suggesting that senescent cells can promote immune evasion of tumors 12. However, the potential mechanism and functional mediator remain unclear.

In order to improve the current proofs and further reveals, the present study aimed to identify key factors as CRC predictor, which potentially mediates cellular senescence and immune evasion. Hopefully, this study can provide a new 'bridge' between cellular senescence and tumor immunity research.

## Results

### Identification of DEGs in CRC and cellular senescence

**Figure 1:**
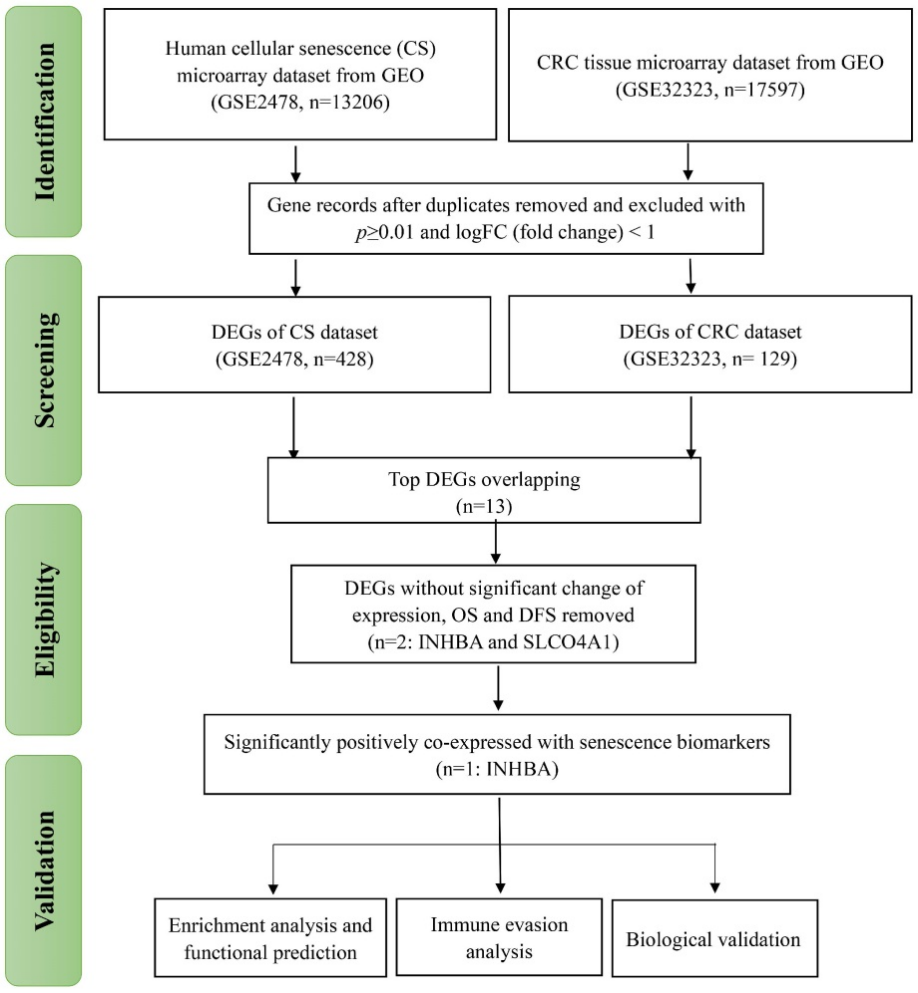
PRISMA flow of the present study. The research procedures of this study were classified into four parts: 'Identification', 'Screening', 'Eligibility' and 'Validation'.

**Figure 2:**
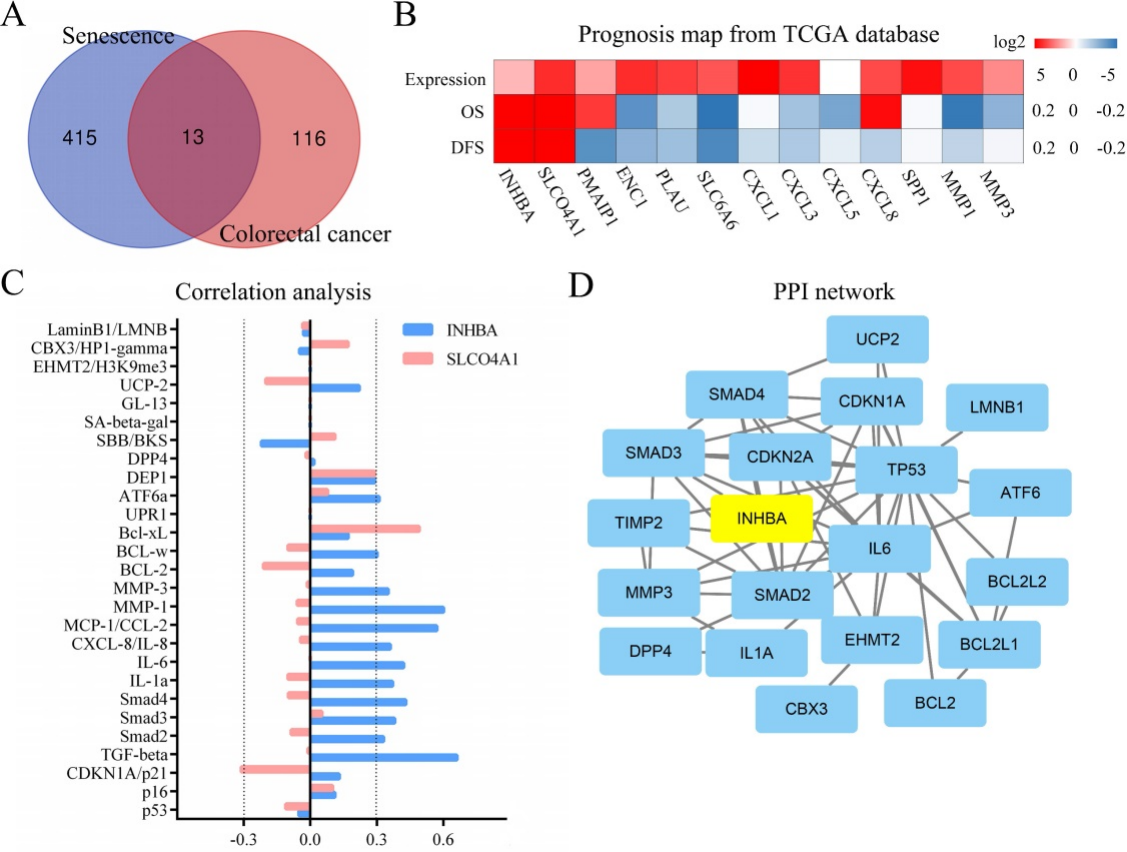
** (A)** DEGs were selected with a fold‑change >2 and P<0.01 in the mRNA expression profiling datasets of GSE2478 (Oncogene-induced senescence) and GSE32323 (Colorectal cancer tumors). The overlapping analysis were conducted. **(B)** 13 DEGs were assessed with expression, OS and DFS from TCGA. The related indexes were marked in colors [high (red) to low (blue)] and displayed as a heatmap (p<0.05). **(C)** Correlation among INHBA, SLCO4A1 and senescence biomarkers were respectively analyzed with Spearman and the correlation indexes were converged in a histogram (p<0.05). **(D)** The protein-protein interacting network for INHBA and senescence biomarkers was generated (negative interacting proteins were excluded).

As displayed in Figure 1, the total analytical processes of this study were listed in the PRISMA flow. Two datasets (GSE2478: Oncogene-induced senescence; GSE32323: Colorectal cancer tumors) were obtained from the GEO database and included in the present study. GSE2478 and GSE32323 were respectively analyzed with GEO2R. Genes with log Fold Change (FC) >2 and p<0.01 were chosen as DEGs. Overlap analyses were conducted and 13 DEGs (INHBA, SLCO4A1, PMAIP1, ENC1, PLAU, SLC6A6, CXCL1, CXCL3, CXCL5, CXCL8, SPP1, MMP1, MMP3) were screened (Fig. 2A). Via gene expression, overall survival (OS), disease free survival (DFS) of TCGA database, INHBA and SLCO4A1 were found overexpressed in tumor tissues and significantly OS-/DFS- differential (Fig. 2B). To determine the senescence pertinence, 9 bio-processes of cellular senescence and 27 relative biomarkers were picked out according to literature. GEPIA online tool was introduced to perform correlation analyses among INHBA, SLCO4A1 and 27 senescence biomarkers (Table 1). As Figure 2C shown, INHBA was positively co-expressed with most senescence biomarkers. Conversely, SLCO4A1 was negatively co-expressed with most of them. The PPI network was generated from INHBA and 19 senescence related biomarkers (other 8 showed no interaction with INHBA) (Fig. 2D). Results showed that INHBA was screened out as both a CRC predictor and senescence related factor.

### Prognostic meta-analysis and enrichment analysis of INHBA

**Figure 3:**
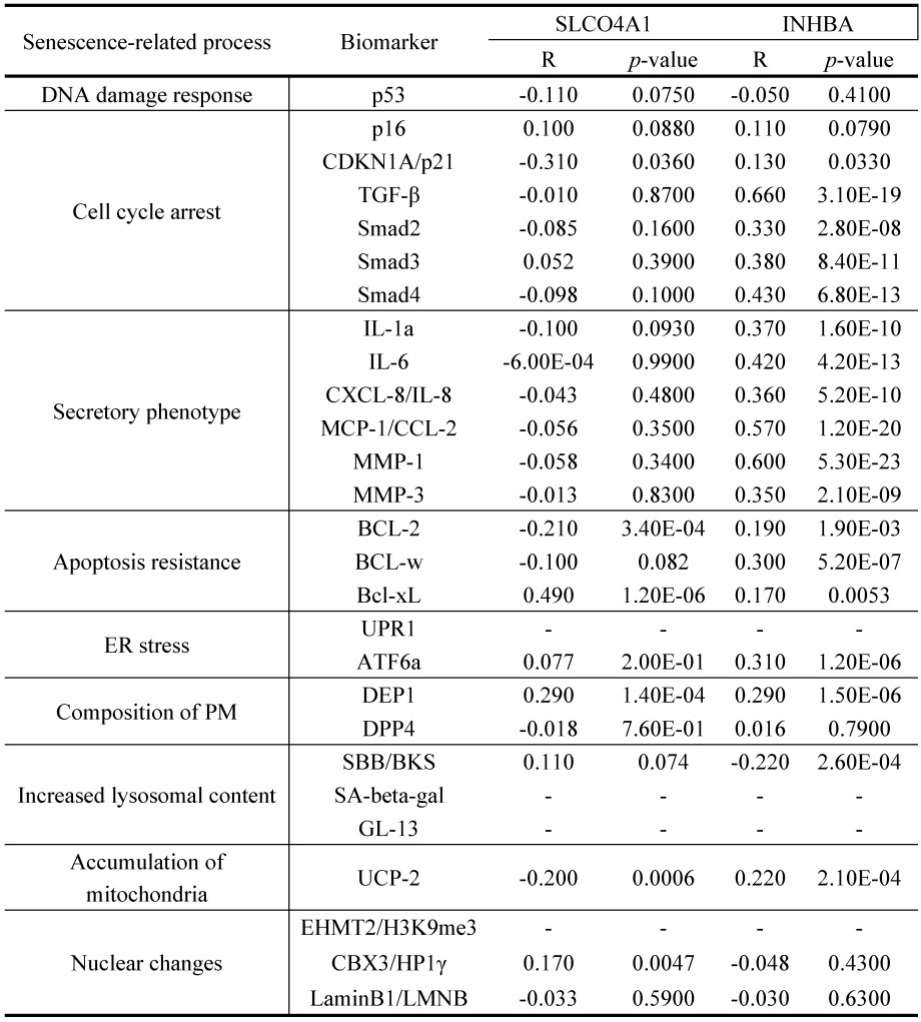

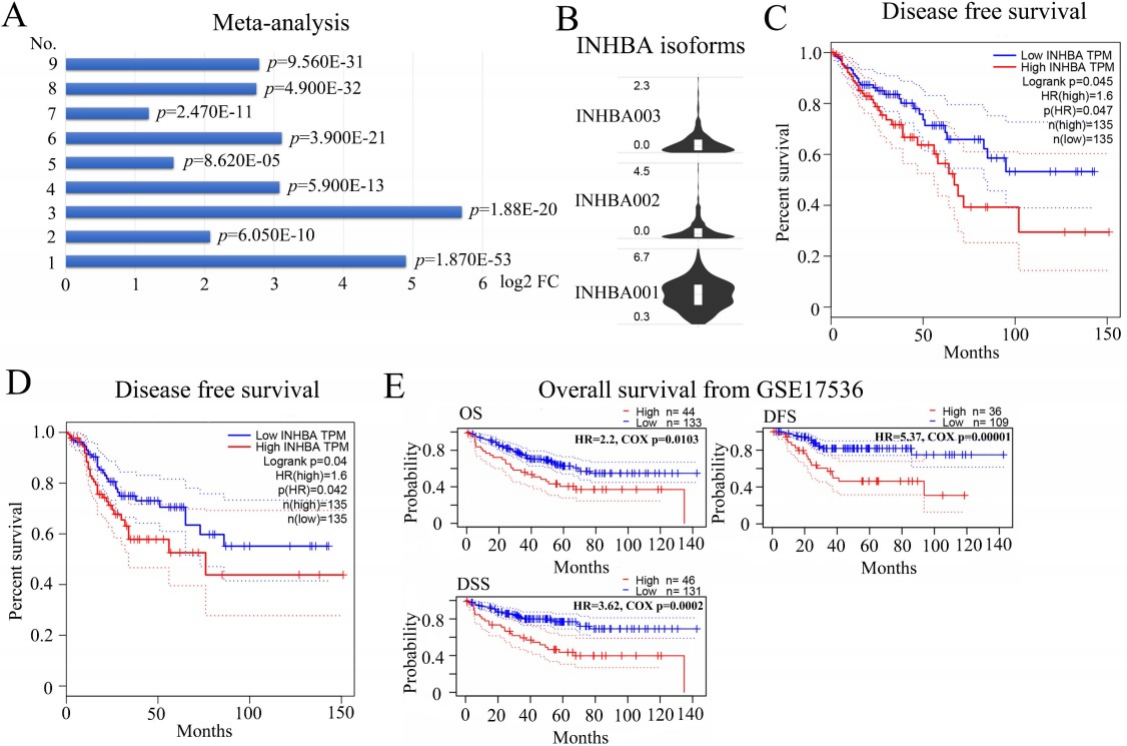
** (A)** Meta‑analysis of MUC4 expression levels was performed with 9 datasets. The log2 FC indexes in a histogram (p<0.05). **(B)** Relative mRNA expression of INHBA isoforms in CRC from TCGA. **(C)** OS and DFS of INHBA were analyzed from TCGA and generated with the Kaplan-Meier curve (p<0.05). **(E)** OS, DFS and DSS of INHBA were obtained and analyzed from GSE17536 and generated with the Kaplan-Meier curve (p<0.05).

**Figure 4:**
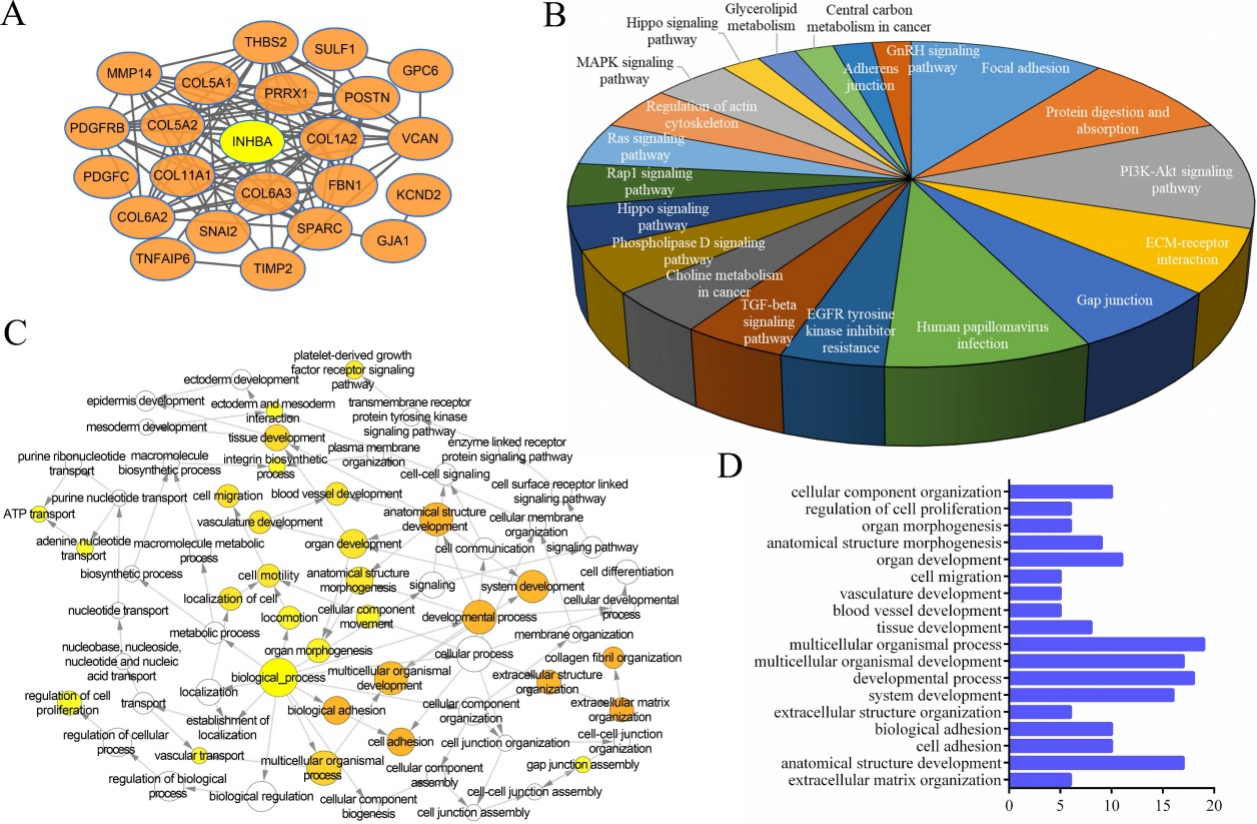
** (A)** PPI network was generated among INHBA and top similar genes from TCGA in CRC. **(B)** KEGG and **(C)** GO of similar gene set were enriched. **(D)** Top related bio-processes were listed in a histogram.

A multiple meta-analysis was made from published data in Oncomine online database. In 9 CRC datasets, INHBA was substantially overexpressed in tumor tissues (log2 FC > 2) (Fig. 3A). Expressions of INHBA in 275 normal colorectal tissues and 349 tumor tissues were obtained from TCGA. Expressions of INHBA isoforms (INHBA001, INHBA002, INHBA3) were analyzed from TCGA and INHBA001 was relatively overexpressed compared with other two isoforms (Fig. 3B). Kaplan-Meier curves were conducted to analyze the OS and DFS from TCGA (Fig. 3C and 3D). Meanwhile, OS, DFS and disease specific survival (DSS) were analyzed from the GEO database (GSE17536) (Fig. 2E). Overexpression of INHBA was determined significantly correlated with the decline of CRC patients' survival time. Top similar genes with INHBA were obtained from GEPIA (Table 2) and PPI network was generated (Fig. 4A). Enrichment of KEGG, bio-process interaction and GO terms were established. Results showed that INHBA was mainly enriched in focal adhesion, PI3K-Akt signaling pathway and ECM-receptor (Fig. 4B). Meanwhile, INHBA was mainly enriched in biological process, multicellular organismal development and developmental process (Fig. 4C and 4D).

### Correlation analysis of immune infiltration and immune evasion

**Figure 5:**
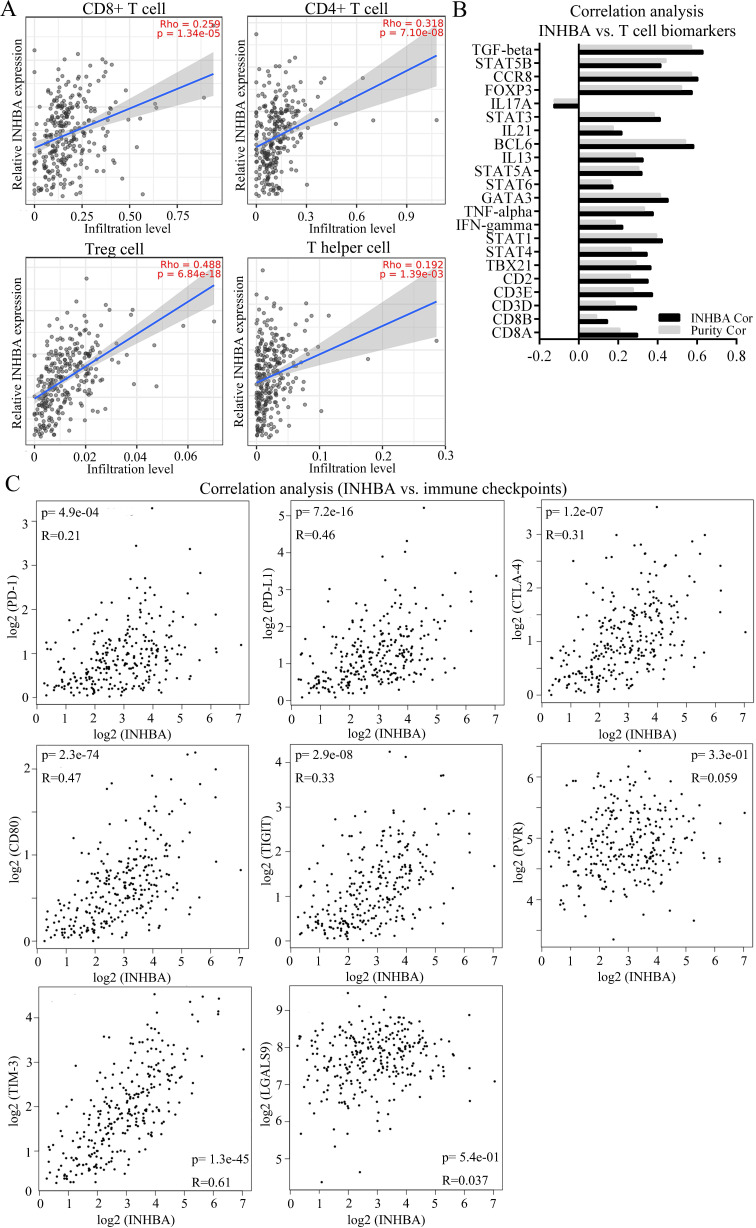
** (A)** Infiltration levels of CD8+ T cell, CD4+ T cell, Treg cell and T helper cell were respectively analyzed and displayed. **(B)** Correlation among INHBA, T cell biomarkers and immune purity were respectively analyzed with Spearman and relative indexes were contained in a histogram (p<0.05). **(C)** Correlation between INHBA and immune checkpoints / -ligands were respectively analyzed with Spearman (p<0.05).

To identify the correlation between INHBA and immunity bio-processes, CIBERSORT method was used to analyze the relative immune infiltration from TIMER. Figure 5A showed that expression of INHBA was positively correlated with T cell (CD8+ T cell, CD4+ T cell, Treg cell and T helper cell) infiltration in CRC. T cell related biomarkers were picked out according to literature (Table 3) and correlation analyses were conducted. Relative correlation indexes of INHBA and purity respectively with T cell biomarkers were listed in Figure 5B. To identify the association with immune checkpoints, immune checkpoints were included and analyzed. Among them, PD-1, PD-L1, CTLA-4, CD80/B7, TIGIT and TIM-3 were discovered positively co-expressed with INHBA (Fig. 5C). Results illustrate that INHBA potentially regulates T cell infiltration and functions, along with which INHBA can mediate immune evasion.

### Validation of INHBA and immune checkpoints in CRC

**Figure 6:**
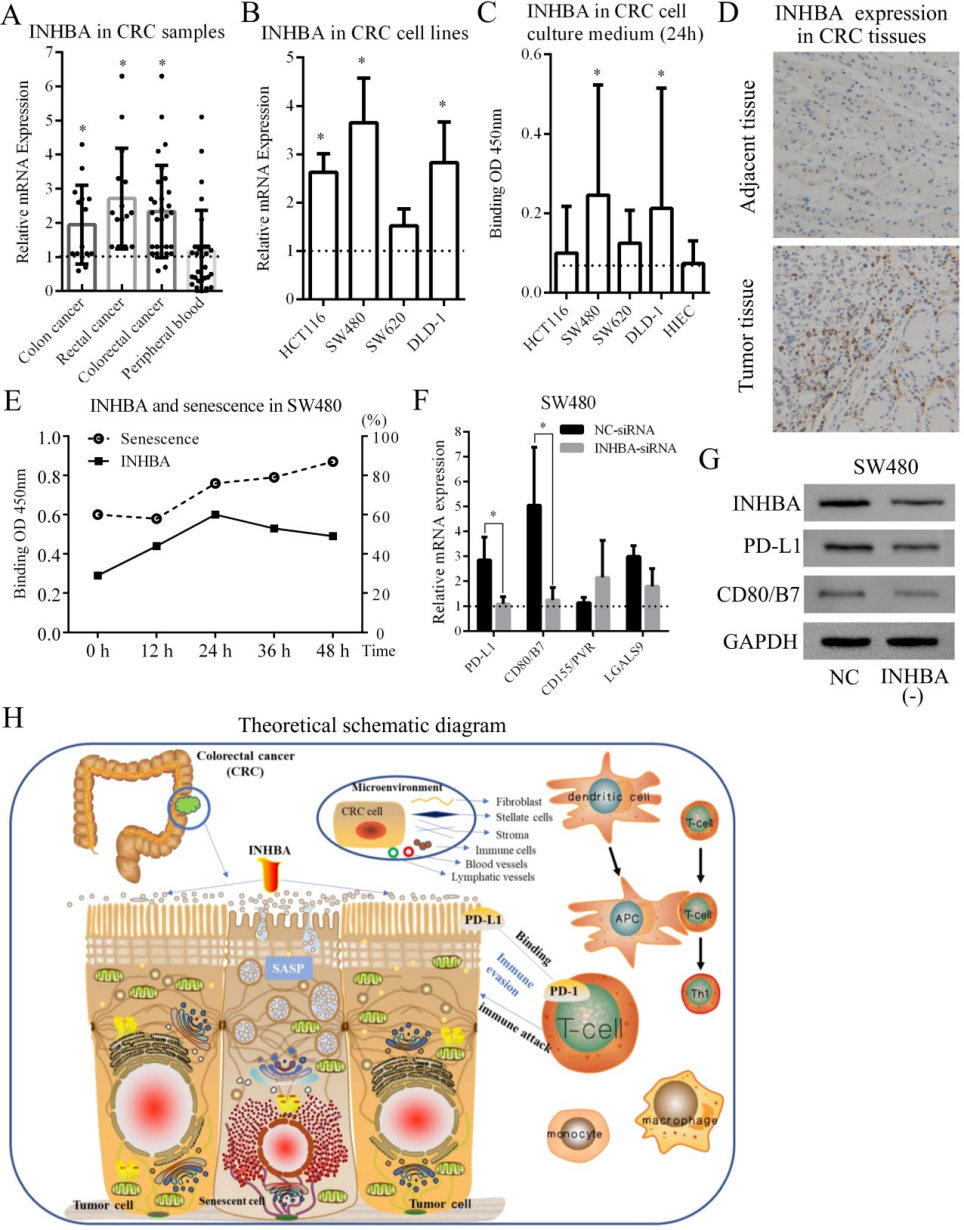
** (A)** Relative INHBA mRNA expression of CRC patients' samples [colon cancer, rectal cancer, colorectal cancer (merged) and peripheral blood] were assessed. **(B)** Relative INHBA mRNA expression of CRC cell lines (HCT116, SW480, SW620 and DLD-1) were assessed. **(C)** INHBA expression in CRC cell culture medium (HCT116, SW480, SW620, DLD-1 and HIEC) were assessed. **(D)** INHBA expression was detected in adjacent tissue and tumor tissue of CRC patients (only display one set here as representative). **(E)** Levels of INHBA and senescence were measured at 0 h, 12 h, 24 h, 36 h and 48 h. **(F)** Relative mRNA expression of immune checkpoints (PD-L1, CD80/B7, CD155/PVR and LGALS9) in shNC/shINHBA SW480 cell lines were assessed. **(G)** Protein expression of PD-L1 and B7 in normal control (shNC) and INHBA (-) (INHBA-shRNA) SW480 cell lines were assessed. **(H)** Biological regulating processes were generated in a theoretical schematic diagram. p<0.05 is labelled as '*'.

30 CRC patients* (*15 with colon cancer and 15 with rectal cancer) and CRC cell lines (HCT116, SW480, SW620 and DLD-1) were selected from the Affiliated Changzhou No. 2 people's hospital of Nanjing medical university, who received tumorectomy (Table 4). Tissue samples and peripheral blood samples were obtained from patients and INHBA mRNA expressions were measured by qRT-PCR. Figure 6A shows that INHBA were overexpressed in colon, rectal and combined (colorectal) cancer tissues compared with adjacent tissues. However, no significant change was found in peripheral blood samples. In 4 CRC cell lines, INHBA was overexpressed compared with normal colonic epithelial cell line (HCoEpiC) (Fig. 6B). Meanwhile, expressions of INHBA in cell culture medium were measured by ELISA. INHBA was dramatically overexpressed in SW480 and DLD-1 (Fig. 6C). As shown in Figure 6D, INHBA expressions in CRC patients' tumor and adjacent tissues were quantified by IHC. Accordingly, SW480 cell line was selected for senescence induction assay. With 5-FU treatment, senescence levels increased with time. In cell medium, INHBA levels were found elevated in 0 - 24h and slightly decline in 24 - 48h (Fig. 6E). siRNA was used to interfere INHBA expression in SW480. mRNA expressions of immune checkpoints were detected in INHBA (-) and normal control SW480. Expressions of PD-L1 and CD80/B7 were found dramatically decreased in INHBA (-) SW480 compared with normal control SW480 (Fig. 6F). In addition, protein levels of INHBA, PD-L1 and CD80/B7 were measured INHBA (-) and normal control SW480. INHBA, PD-L1 and CD80/B7 were found decreased in INHBA (-) SW480 compared with normal control SW480 (Fig. 5G). Results suggest that INHBA was overexpressed in CRC tumor samples, cell lines and culture medium compared with normal ones. Moreover, mutation of INHBA leads to expression decline of PD-L1 and CD80/B7, which are established to be ligands of PD-1 and CTLA-4.

## Discussion

CRC is one of the most common gastrointestinal (GI) cancers. The immune system in the colon and rectum is tuning all the time to maintain the balance of self-tissue regeneration and immunity against potential pathogens. Disruption of normal immune homeostasis and excessive inflammation may lead to autoimmunity diseases, including inflammatory bowel disease and cancers 13. In contrast, normal immunosurveillance is critical to eliminate potential pathogens and eradicate sporadic tumorigenic cells to prevent GI diseases, including CRC. Theoretically, the immune system distinguishes autogenous from non-autogenous cells by binding T cell receptors (TCR) on T cells to polypeptide complexes of major histocompatibility complex (MHC) class I molecules on the surfaces, including tumor cells. The TCR-MHC binding bio-processes are regulated by co-stimulation or co-inhibition of signals that contribute tumor cells to evade immune attack. In recent years, inhibitors of immune check points have become a hot topic in the tumor immunotherapy research 14-16.

SASP is an independent senescent phenotype in cell, which can secrete innumerable inflammatory, extracellular modifying and growth factors impacting on tumorigenesis. In some cancer models (such as liver fibrosis and hepatocellular carcinoma, HCC), SASP factors are derived from senescent stroma in early stages, which are competent to limit tumor growth by eliciting the immune response. However, SASP plays differential roles in tumors. When SASP was induced in the terminal stages of malignancy in HCC, it created an immunosuppressive environment that promoted tumor proliferation. SASP-mediated immunosuppressive environments are not confined to advanced stages of liver tumorigenesis 17, 18. Ruhland, M. K. et al demonstrated that SASP mediating senescent fibroblasts in a skin model that significantly inhibited CD8+ T cell activity by suppressive immunocytes recruitment, suggesting that SASP and senescent cells can elicit immunosuppressive environments that promote tumor cell growth 19, 20. Generally, SASP is considered mediating immune environment through regulation of TGF-β, NOTCH, JAK/STAT pathways. However, mechanisms and functional networks remain unclear.

INHBA is a member of the TGF superfamily. In human cells, INHBA is encoded in the cellular nucleus, synthesized in the cytoplasm and secreted through the membrane. Studies showed that overexpression of INHBA is positively correlated with poor prognosis in esophageal, prostate and ovarian cancer. What's more, the expression of INHBA is significantly associated with tumor lymph node metastasis (TNM) stage 21-24. Thus, INHBA potentially serves as an independent prognostic factor for GI cancers. In this present study, DEGs were screened out from microarray datasets of human senescent cells and CRC cells. Through prognostic analysis, functional enrichment and signaling pathway prediction, INHBA was recognized correlating with poor prognosis of CRC patients, as well as mediating cellular senescence in CRC cells. Besides, INHBA was found significantly correlated with immune infiltration, especially T cells, which implied that INHBA might involve tumor immune regulation 25. As we know, recruitment of immunosuppressive cells (i.e. Tregs) is a critical process leading to immune evasion. Then, the results of correlation analyses showed that INHBA was co-expressed with either multiple T cell biomarkers or common immune checkpoints (PD-1, PD-L1, CTLA-4, CD80/B7, TIGIT, CD155/PVR, TIM-3, LGALS9). In biological validation, INHBA appeared over-expressed in human CRC tissues and cell lines, compared with normal controls. After senescence induction in SW480, INHBA level in the medium increased with senescence level. The levels of INHBA slightly decreased after 24h, possibly owing to the partial cell death. In addition, expressions of PD-L1 and CD80/B7 were revealed significant decline in INHBA-mutated SW480 comparing with normal control. These results demonstrated that INHBA acted as a prognostic predictor in CRC and a regulator of cellular senescence and immune checkpoint ligands (PD-L1 and CD80/B7) in CRC. Potentially, INHBA could be overexpressed and secreted CRC senescent cells. Secreted INHBA is capable to regulate both neighbor CRC cells and microenvironment, which lead to immune evasion. Accordingly, we generate a hypothesis diagram (Fig. 5H).

In conclusion, INHBA is a novel protein regulating cellular senescence and immune evasion in colorectal cancer. Interactions of INHBA between cellular senescence and immune evasion has been indicated feasible and ponderable, but still require more proofs. In an on-going study, we intend to expose the regulating interactions among INHBA, TGF-β family proteins and immune checkpoint ligands (mainly on PD-L1 and CD80/B7). Since ICIs have been applied in patients, we hope this study will provide new approaches for enhancement and anti-resistance of Immunotherapy.

## Materials and methods

### Cell culture

The human CRC cell lines (HCT116, SW480, SW620, DLD-1) and the human normal colonic epithelial cell line (HIEC) as well as tool cell line (293T) were purchased from the Cell Resource Center of Shanghai Institute of Biochemistry and Cell Biology (The Chinese Academy of Sciences). The cells were cultured in MCCOY'5A medium (Gibco; Thermo Fisher Scientific, Inc.) supplemented with 10% FBS (Gibco; Thermo Fisher Scientific, Inc.) and maintained at 37 °C with 5% CO2.

### Patient studies

Between January 2020 and July 2020, a total of 30 CRC patients (15 with colon patients and 15 with rectal cancer) were recruited at the Affiliated Changzhou No. 2 People's Hospital of Nanjing Medical University (Changzhou, China). The patients were diagnosed with CRC by colonoscope examination, biopsy, abdominal CT scan and surgery with clinical standards. Tumor stages of CRC were systematically assessed with TNM classification. Other including criteria: a. Adult but age<70; b. without adjuvant chemotherapy or other anticancer therapy; c. without serious underlying diseases and general familial-hereditary diseases; d. without partial or systemic immune disorders.

The study was approved by the Ethics Committee of the Affiliated Changzhou No. 2 People's Hospital of Nanjing Medical University [approval no: (2019) KYO 073‑01], and all patients provided informed consent. PC and adjacent tissue samples (>5 cm from the tumor) were obtained during surgery. Peripheral blood (PB) samples were obtained before surgery. All samples were stored at ‑80 °C and preprocessed for further research.

### Analysis of Microarrays and DEGs

Two datasets [GSE2478: Oncogene-induced senescence; GSE32323: Colorectal cancer tumors] were downloaded from the Gene Expression Omnibus (GEO) database (http://www.ncbi.nlm.nih.gov/geo) 26. The DEGs between the tumor and control groups were identified using the GEO2R webtool (http://www.ncbi.nlm.nih.gov/geo/geo2r) 27 by comparing the GEO microarray datasets. Probes without corresponding gene symbols or genes with redundant probe sets were eliminated or processed using the DAVID online tool (https://david.ncifcrf.gov/) 28 respectively. A log [fold‑change (FC)] >2 and P<0.01 were selected to identify statistically significant differences. The overlap analysis of DEGs from the OIS and CRC datasets was conducted and displayed in Venn diagrams (http://bioinformatics.psb.ugent.be/webtools/Venn/) 29.

### Clinical analysis of TCGA and enrichment analysis

Kyoto Encyclopedia Genes and Genomes (KEGG) Orthology Based Annotation System (KOBAS 3.0; http://kobas.cbi.pku.edu.cn/kobas3) web server was used for gene/protein functional annotation and functional set enrichment in the present study. Gene Ontology (GO) functional term enrichment analysis tool was used to determine gene functions and perform biological analysis 30. KEGG signaling pathway enrichment analysis was used to illustrate gene functions and biological pathways 31. P<0.05 was considered to indicate a statistically significant difference. Gene expression data of CRC were acquired from The Cancer Genome Atlas (TCGA) database (https://portal.gdc.cancer.gov) 32. Overall Survival (OS) and Disease‑Free Survival (DFS) analyses of patients grouped into high/low INHBA mRNA expression groups based on the median expression levels were performed using the Gene Expression Profiling Interactive Analysis (GEPIA) online database (https://gepia.cancer‑pku.cn) 33. The associations between expression levels and the meta‑analysis of data from 7 previous studies 34-39 were analyzed using the Oncomine database (https://www.oncomine.com) 40.

### Reverse transcription‑quantitative PCR (RT-qPCR)

Total RNA was extracted from the PC cell line and tissues, as well as the corresponding controls, using TRIzol® reagent (Invitrogen; Thermo Fisher Scientific, Inc.). The total RNA was reverse‑transcribed into cDNA using a PrimeScript™ RT reagent kit (Takara Biotechnology Co., Ltd.). qPCR was subsequently performed using a SYBR® Premix Ex Taq kit (Takara Biotechnology Co., Ltd.) on a 7500 Real‑Time PCR system (Applied Biosystems; Thermo Fisher Scientific, Inc.). The reverse transcription was performed at 42 °C for 1 h, followed by 95 °C for 5 min. The thermocycling conditions were as follows: Initial denaturation step at 95 °C for 10 min, followed by 40 cycles at 95 °C for 15 sec and at 60 °C for 1 min. The expression levels were calculated using the 2^‑ΔΔCq^ method 41. The primer pairs used were as following: INHBA forward, 5′-ACACAACAACTTTTGCTGCC-3′; reverse, 5′-TCGTGTCACCA CTGTCTTCTC-3′. PD-L1 forward: 5′-GGAGATTAGATCCTGAGGAAAACCA-3′; reverse: 5′-AACGGAAGATGAATGTCAGTGCTA-3′. CD80/B7 forward: 5ʹ- CTAGCATAAAGCCATTTAA AGAGGT-3ʹ; reverse: 5ʹ-TTATTGGTGTTTACCCAGTATTCC-3'. CD155/PVR forward: 5′-GCTAGAAGGACTCACTAGACTCAGGAA-3′; reverse: 5′-GTCGCCTCATCTGTCGTGGAAC-3′. LGALS9 forward: 5'- CGTCAATGGCTCTGTGCAGCTGTC-3'; reverse: 5'- AGATCCACAC TGAGAAGCTCTGGC-3'. GAPDH forward, 5'‑ATCATCCCTGCCTCTACTGG‑3'; reverse, 5'‑GTCAGG TCCACCACTGACAC‑3'.

### Short hairpin RNA transfection

Short hairpin RNAs (shRNAs) targeting INHBA (shINHBA) and transduction & transfection kits were produced by RiboBio (Guangzhou, China). Constructed plasmids (with anti-puromycin) were transfected and packaged into 293T. Lentivirus was harvested and transfer to target cell (SW480). Transfected target cells were incubated at 37 °C with 5% CO2 for 24 h and observed every 8 h. Transfected target cells were treated with Puromycin (Sigma-Aldrich, USA) (250 ng/ml) for 3 days for screening. The negative control shRNA (shNC) was used to determine the effects of shRNA delivery. All the shRNA plasmids were provided by RiboBio. INHBA knockdown efficiency was determined by RT-qPCR.

### Western blot analysis

SW480 cells were incubated and lysed on ice for 40 min. Protein was extracted and the concentrations were measured using the Bradford assay. Lysates were then separated by 12% SDS-polyacrylamide gel electrophoresis, transferred to polyvinylidene difluoride membranes (Sigma-Aldrich, USA) and incubated with the indicated antibodies. Autoradiograms were quantified by densitometry (Quantity One software; Bio-Rad, Hercules, California, USA). An anti-GAPDH antibody was used as a control. Anti-INHBA, anti-PD-L1, anti-CTLA-4 and anti-GAPDH were purchased from Cell Signaling Technology, Inc (Beverly, Massachusetts, USA).

### ELISA analysis

Cell culture medium solutions were centrifuged at 3000 × g for 30 min at 10 °C in the centrifuge. The aqueous layer was decanted, and pellets and fat layers were discarded. Samples were frozen at -20 °C until the time of analysis. INHBA was determined with ELISA kit (R&D Systems, Minneapolis, MN, USA), respectively, according to the manufacturer's instruction.

### Cellular senescence assay

SW480 cell lines were divided into 5 groups and treated by 5-FU (Sigma-Aldrich, USA) at the concentration of 1.5 ug/ml for 48h. 5 groups were respectively harvested with at time of 0 h, 12 h, 24 h and 48 h (mediums were prepared for ELISA assessment). Cells were washed and incubated with SA-β-Clalactosidase solution (Sigma-Aldrich, USA). Stained cell lines were observed by microscopy (20X). The percentage of senescence was measured by [blue-dyed cells numbers (senescent cells)] / [non-dyed cells (non-senescent cells) X 100%] in a microscopic view. Average level of 4 view areas was summarized as the relative senescence level.

### Western blot

SW480 cells were incubated and lysed on ice for 40 min. Protein was extracted and the concentrations were measured using the Bradford assay. Lysates were then separated by 12% SDS-polyacrylamide gel electrophoresis, transferred to polyvinylidene difluoride membranes (Sigma-Aldrich) and incubated with the indicated antibodies. Autoradiograms were quantified by densitometry (Quantity One software; Bio-Rad, Hercules, California, USA). An anti-GAPDH antibody was used as a control. Anti-INHBA, anti-PD-L1, anti-CD80 antibodies were purchased from Cell Signaling Technology, Inc (Beverly, Massachusetts, USA).

### Statistical analysis

Data are presented as the mean ± SEM. SPSS 17.0 (SPSS statistics, IBM Inc.) was used for statistical analysis. GraphPad Prism 6.0 software (GraphPad Software, Inc.) was used to generate the diagrams. Statistical differences were determined using one‑way ANOVA with the Bonferroni post hoc test for multiple comparisons. Correlation analysis was performed using the Spearman correlation coefficient. P<0.05 was considered to indicate a statistically significant difference.

## Figures and Tables

**Table 1:** Correlation between INHBA and senescence related biomarkers

**Table 2:**
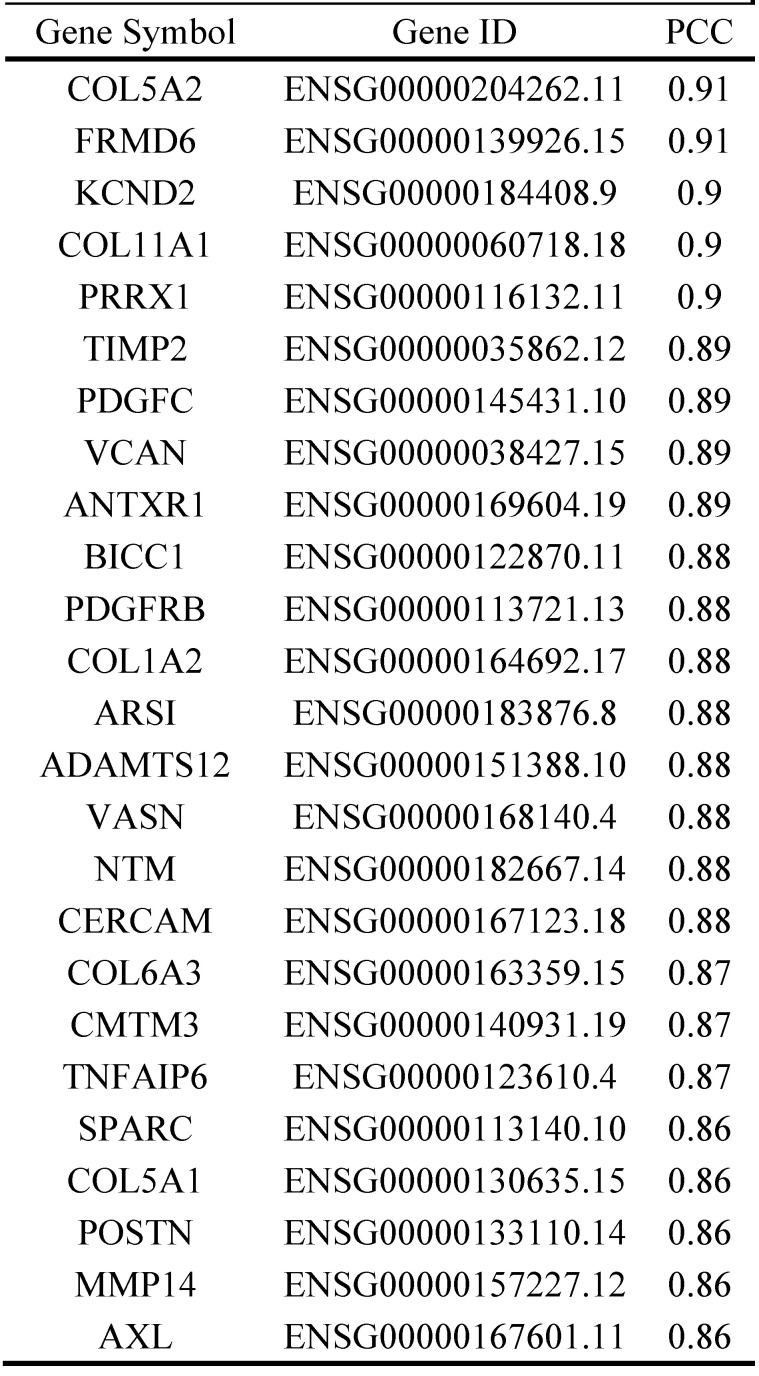
Top 25 similar genes to INHBA in CRC

**Table 3:**
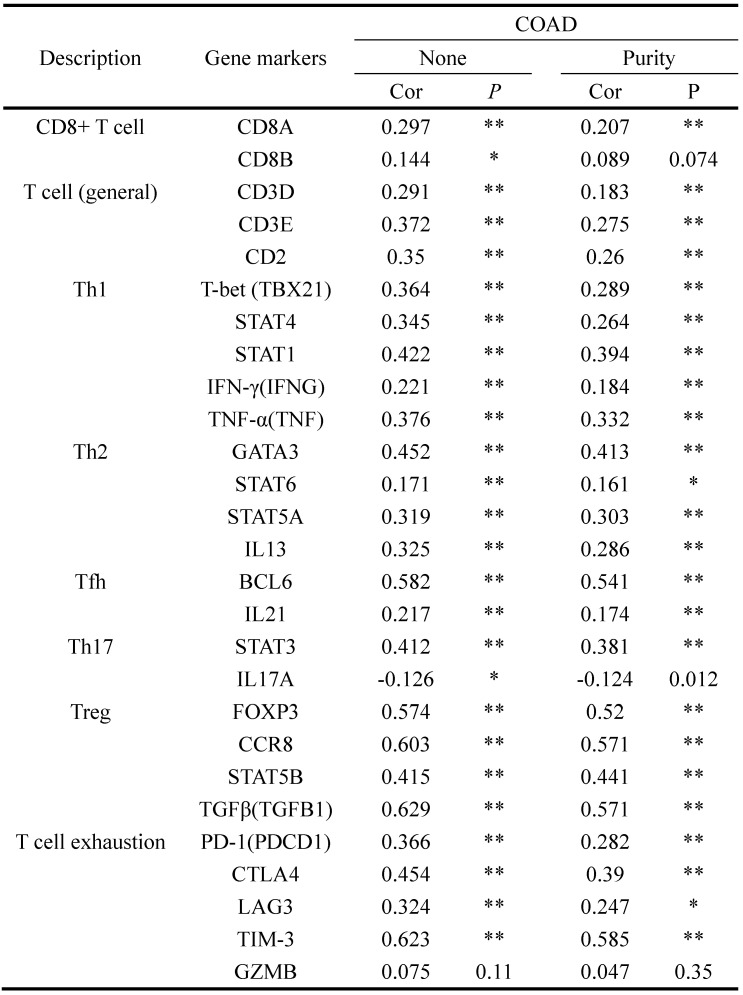
Correlation of INHBA and T cell biomarkers

'*': p<0.1; '**': p<0.05.

**Table 4:**
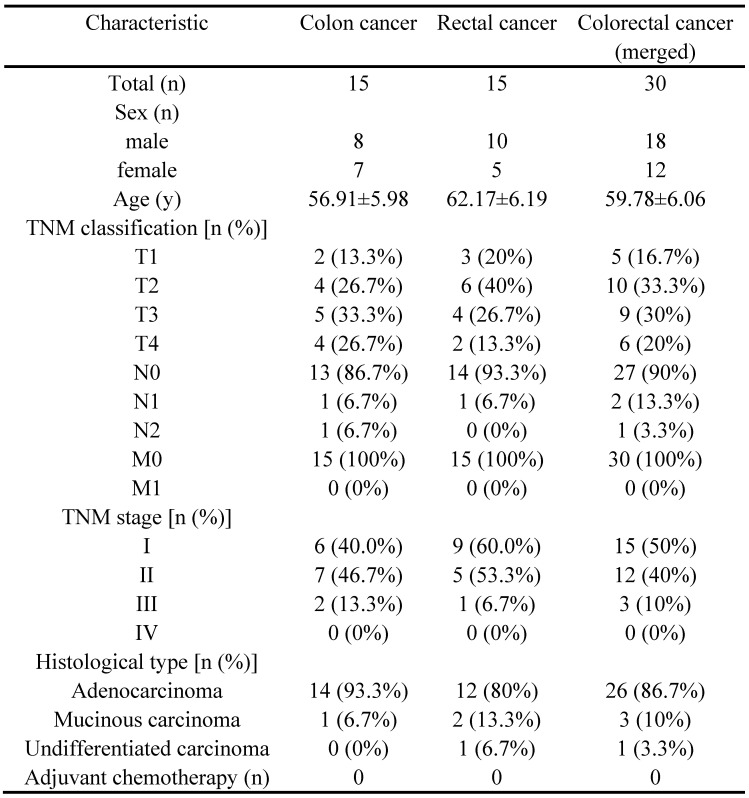
Clinicopathological characteristics of patients

## Abbreviations

CRC: Colorectal Cancer
OIS: Oncogene Induced Senescence
GEO: Gene Expression Omnibus
DEGs: Differentially Expressed Genes
TCGA: The Cancer Genome Atlas
siRNA: Small Interfering RNA
INHBA: Inhibin Beta A
TCR: T Cell Receptors
MHC: Major Histocompatibility Complex
ICIs: Immune Checkpoint Inhibitors
PD-1/PD-L1: Programmed Death protein-1/ligand-1
CTLA-4: Cytotoxic T Lymphocyte Antibody 4
SASP: Senescence Associated Secretion Phenotype
OS: Overall Survival
DFS: Disease Free Survival
DSS: Disease Specific Survival
FC: Fold‑Change
KEGG: Kyoto Encyclopedia Genes and Genomes
GO: Gene ontology
GEPIA: Gene Expression Profiling Interactive Analysis
PPI: Protein - Protein Interacting

## References

[1] Dekker E, Tanis PJ, Vleugels JLA, Kasi PM, Wallace MB (2019). Colorectal cancer. The Lancet.

[2] Siegel RL, Miller KD, Jemal A (2020). Cancer statistics, 2020. CA: a cancer journal for clinicians.

[3] Martini G, Troiani T, Cardone C, Vitiello P, Sforza V, Ciardiello D (2017). Present and future of metastatic colorectal cancer treatment: A review of new candidate targets. World journal of gastroenterology.

[4] Vinay DS, Ryan EP, Pawelec G, Talib WH, Stagg J, Elkord E (2015). Immune evasion in cancer: Mechanistic basis and therapeutic strategies. Semin Cancer Biol.

[5] Ganesh K, Stadler ZK, Cercek A, Mendelsohn RB, Shia J, Segal NH (2019). Immunotherapy in colorectal cancer: rationale, challenges and potential. Nature reviews Gastroenterology & hepatology.

[6] Tintelnot J, Stein A (2019). Immunotherapy in colorectal cancer: Available clinical evidence, challenges and novel approaches. World journal of gastroenterology.

[7] Rhinn M, Ritschka B, Keyes WM (2019). Cellular senescence in development, regeneration and disease. Development (Cambridge, England).

[8] Tchkonia T, Zhu Y, van Deursen J, Campisi J, Kirkland JL (2013). Cellular senescence and the senescent secretory phenotype: therapeutic opportunities. The Journal of clinical investigation.

[9] Faget DV, Ren Q, Stewart SA (2019). Unmasking senescence: context-dependent effects of SASP in cancer. Nature reviews Cancer.

[10] Prieto LI, Baker DJ (2019). Cellular Senescence and the Immune System in Cancer. Gerontology.

[11] Wu Q, Li B, Liu L, Sun S, Sun S (2020). Centrosome dysfunction: a link between senescence and tumor immunity. Signal Transduct Target Ther.

[12] Saleh T, Tyutyunyk-Massey L, Gewirtz DA (2019). Tumor Cell Escape from Therapy-Induced Senescence as a Model of Disease Recurrence after Dormancy. Cancer research.

[13] Lucas C, Barnich N, Nguyen HTT (2017). Microbiota, Inflammation and Colorectal Cancer. International journal of molecular sciences.

[14] Basile D, Garattini SK, Bonotto M, Ongaro E, Casagrande M, Cattaneo M (2017). Immunotherapy for colorectal cancer: where are we heading?. Expert opinion on biological therapy.

[15] Koi M, Carethers JM (2017). The colorectal cancer immune microenvironment and approach to immunotherapies. Future oncology (London, England).

[16] Kreidieh M, Mukherji D, Temraz S, Shamseddine A (2020). Expanding the Scope of Immunotherapy in Colorectal Cancer: Current Clinical Approaches and Future Directions. BioMed research international.

[17] Loo TM, Kamachi F, Watanabe Y, Yoshimoto S, Kanda H, Arai Y (2017). Gut Microbiota Promotes Obesity-Associated Liver Cancer through PGE(2)-Mediated Suppression of Antitumor Immunity. Cancer discovery.

[18] Yoshimoto S, Loo TM, Atarashi K, Kanda H, Sato S, Oyadomari S (2013). Obesity-induced gut microbial metabolite promotes liver cancer through senescence secretome. Nature.

[19] Ruhland MK, Coussens LM, Stewart SA (2016). Senescence and cancer: An evolving inflammatory paradox. Biochimica et biophysica acta.

[20] Ruhland MK, Loza AJ, Capietto AH, Luo X, Knolhoff BL, Flanagan KC (2016). Stromal senescence establishes an immunosuppressive microenvironment that drives tumorigenesis. Nat Commun.

[21] Li X, Yu W, Liang C, Xu Y, Zhang M, Ding X (2020). INHBA is a prognostic predictor for patients with colon adenocarcinoma. BMC cancer.

[22] Lyu S, Jiang C, Xu R, Huang Y, Yan S (2018). INHBA upregulation correlates with poorer prognosis in patients with esophageal squamous cell carcinoma. Cancer management and research.

[23] Okano M, Yamamoto H, Ohkuma H, Kano Y, Kim H, Nishikawa S (2013). Significance of INHBA expression in human colorectal cancer. Oncol Rep.

[24] Oshima T, Yoshihara K, Aoyama T, Hasegawa S, Sato T, Yamamoto N (2014). Relation of INHBA gene expression to outcomes in gastric cancer after curative surgery. Anticancer research.

[25] Sun YL, Zhang Y, Guo YC, Yang ZH, Xu YC (2020). A Prognostic Model Based on the Immune-related Genes in Colon Adenocarcinoma. International journal of medical sciences.

[26] Clough E, Barrett T (2016). The Gene Expression Omnibus Database. Methods in molecular biology (Clifton, NJ).

[27] Barrett T, Wilhite SE, Ledoux P, Evangelista C, Kim IF, Tomashevsky M (2013). NCBI GEO: archive for functional genomics data sets-update. Nucleic acids research.

[28] Dennis G Jr, Sherman BT, Hosack DA, Yang J, Gao W, Lane HC (2003). DAVID: Database for Annotation, Visualization, and Integrated Discovery. Genome biology.

[29] Luick B (2015). Venn Diagrams: JNEB Figures Research. Journal of nutrition education and behavior.

[30] The Gene Ontology Resource (2019). 20 years and still GOing strong. Nucleic acids research.

[31] Kanehisa M, Goto S (2000). KEGG: kyoto encyclopedia of genes and genomes. Nucleic acids research.

[32] Wang Z, Jensen MA, Zenklusen JC (2016). A Practical Guide to The Cancer Genome Atlas (TCGA). Methods in molecular biology (Clifton, NJ).

[33] Tang Z, Li C, Kang B, Gao G, Li C, Zhang Z (2017). GEPIA: a web server for cancer and normal gene expression profiling and interactive analyses. Nucleic acids research.

[34] Gaedcke J, Grade M, Jung K, Camps J, Jo P, Emons G (2010). Mutated KRAS results in overexpression of DUSP4, a MAP-kinase phosphatase, and SMYD3, a histone methyltransferase, in rectal carcinomas. Genes, chromosomes & cancer.

[35] Graudens E, Boulanger V, Mollard C, Mariage-Samson R, Barlet X, Grémy G (2006). Deciphering cellular states of innate tumor drug responses. Genome biology.

[36] Hong Y, Downey T, Eu KW, Koh PK, Cheah PY (2010). A 'metastasis-prone' signature for early-stage mismatch-repair proficient sporadic colorectal cancer patients and its implications for possible therapeutics. Clinical & experimental metastasis.

[37] Kaiser S, Park YK, Franklin JL, Halberg RB, Yu M, Jessen WJ (2007). Transcriptional recapitulation and subversion of embryonic colon development by mouse colon tumor models and human colon cancer. Genome biology.

[38] Ramaswamy S, Tamayo P, Rifkin R, Mukherjee S, Yeang CH, Angelo M (2001). Multiclass cancer diagnosis using tumor gene expression signatures. Proceedings of the National Academy of Sciences of the United States of America.

[39] Skrzypczak M, Goryca K, Rubel T, Paziewska A, Mikula M, Jarosz D (2010). Modeling oncogenic signaling in colon tumors by multidirectional analyses of microarray data directed for maximization of analytical reliability. PloS one.

[40] Rhodes DR, Yu J, Shanker K, Deshpande N, Varambally R, Ghosh D (2004). ONCOMINE: a cancer microarray database and integrated data-mining platform. Neoplasia (New York, NY).

[41] Singh C, Roy-Chowdhuri S (2016). Quantitative Real-Time PCR: Recent Advances. Methods in molecular biology (Clifton, NJ).

